# India-United States Dialogue on Traditional Medicine: Toward Collaborative Research and Generation of an Evidence Base

**DOI:** 10.1200/JGO.17.00099

**Published:** 2017-11-16

**Authors:** Jeffrey D. White, Barry R. O’Keefe, Jitendra Sharma, Ghazala Javed, Vid Nukala, Aniruddha Ganguly, Ikhlas A. Khan, Nagi B. Kumar, Hasan Mukhtar, Guido F. Pauli, Larry Walker, Sudha Sivaram, Preetha Rajaraman, Edward L. Trimble

**Affiliations:** **Jeffrey D. White, Barry R. O'Keefe, Aniruddha Ganguly**, **Sudha Sivaram**, **Preetha Rajaraman,** and ****Edward L. Trimble**,** National Cancer Institute, Bethesda, MD; ****Jitendra Sharma**and **Ghazala Javed**,** Government of India; ****Vid Nukala**,** US Embassy, New Delhi, India; ****Ikhlas A. Khan**and **Larry Walke**r,** University of Mississippi, Oxford, MS; ****Nagi B. Kumar**,** Moffitt Cancer Center, Tampa, FL; ****Hasan Mukhtar**,** University of Wisconsin-Madison, Madison, WI; and ****Guido F. Pauli,**** University of Illinois at Chicago, Chicago, IL.

## Abstract

Therapies originating from traditional medical systems are widely used by patients in both India and the United States. The first India-US Workshop on Traditional Medicine was held in New Delhi, India, on March 3 and 4, 2016, as a collaboration between the Ministry of Ayurveda, Yoga and Naturopathy, Unani, Siddha, and Homoeopathy (AYUSH) of the Government of India, the US National Cancer Institute (NCI), National Institutes of Health, and the Office of Global Affairs, US Department of Health and Human Services. It was attended by Indian and US policymakers, scientists, academics, and medical practitioners from various disciplines. The workshop provided an opportunity to open a dialogue between AYUSH and NCI to identify promising research results and potential topics for Indo-US collaboration. Recommendations that emerged from the workshop underlined the importance of applying rational and scientific approaches for drug development; standardizing traditional medicine products and procedures to ensure reliability and reproducibility; promotion of collaboration between Indian traditional medicine practitioners and researchers and US researchers; greater integration of evidence-based traditional medicine practices with mainstream medical practices in India; and development of training programs between AYUSH and NCI to facilitate crosstraining. Several positive developments took place after the thought-provoking deliberations.

## INTRODUCTION

Traditional medicine (TM) is defined by WHO as “the sum total of the knowledge, skills and practices on the basis of the theories, beliefs and experiences indigenous to different cultures, whether explicable or not, used in the maintenance of health, as well as in the prevention, diagnosis, improvement or treatment of physical and mental illnesses.”^1^ Typically, TM is one of the main sources of health care in a country when one or more of the following conditions apply: strong cultural and historical influences, lack of available alternate forms of medicine, or as complementary therapy in addition to other forms of medicine.

India has 15 agroclimatic zones, 47,000 plant species, and 15,000 medicinal plants. This includes approximately 7,000 plants used in Ayurveda, 700 in Unani, 600 in Siddha, and 30 in modern medicine. This makes India one among 12 mega–biodiverse countries of the world.^2^ In rural India, an estimated 65% of the population uses TM to help meet primary health care needs.^3^ The Ministry of Ayurveda, Yoga and Naturopathy, Unani, Siddha, and Homoeopathy (AYUSH) of the Government of India was formed on November 9, 2014, by elevation of the Department of AYUSH, previously under the Ministry of Health and Family Welfare.^4^ The Ministry of AYUSH aims to achieve the following:

- Upgrade the educational standards in Indian Systems of Medicines and Homoeopathy colleges in the country,
- Strengthen existing research institutions and ensure a time-bound research program on identified diseases for which these systems have an effective treatment,
- Draw up schemes for promotion, cultivation, and regeneration of medicinal plants used in these systems, and
- Evolve pharmacopeial standards for Indian Systems of Medicine and Homoeopathy drugs.^5^

As part of the 12th Five-Year Plan (2012 to 2017), the Government of India launched the National AYUSH Mission, which envisages better access to AYUSH services through an increase in the number of AYUSH hospitals and dispensaries; mainstreaming of AYUSH through colocation of AYUSH facilities at primary health centers, community health centers, and district hospitals; and ensuring availability of AYUSH drugs and trained manpower. The plan also aims to improve the quality of AYUSH education through enhancement of the number of upgraded educational institutions, sustained availability of quality raw materials, and promoting medicinal plant conservation.^6^ Of the three pharmacopeia committees for Ayurvedic, Siddha, and Unani drugs, the Pharmacopoeial Laboratory for Indian Medicines, Ghaziabad, has thus far published standards on 1,082 single drugs and 302 compound formulations.^7^ The Ministry of AYUSH has also set up a research portal, managed by the National Institute of Indian Medical Heritage, for disseminating knowledge regarding AYUSH systems and research updates for academic purposes.^8^ The Traditional Knowledge Digital Library was initiated in 2001 as a collaborative project between the Indian Council of Scientific and Industrial Research, the Ministry of Science and Technology, and the then–Department of AYUSH. Managed by the Council of Scientific and Industrial Research, the Traditional Knowledge Digital Library database contains 34 million pages of formatted information on over 0.29 million medicinal formulations of Ayurveda, Unani, Siddha, and asanas of yoga in five international languages (English, German, French, Japanese, and Spanish). Access has been provided to 10 international patent offices under a nondisclosure agreement to check for prior art while granting patents.^9^

According to the 2012 US National Health Interview Survey, one in three adults in the United States used complementary and alternative medicine (CAM) approaches, with natural dietary supplement products and yoga being the most commonly used CAM modalities. It has been estimated that in 2006, Americans spent 33.9 billion US dollars out of pocket on CAM products and services.^10^

Several arms of the US Department of Health and Human Services (HHS) address aspects of TM. Natural products from herbal sources (“botanicals”) are often sold as dietary supplements and are readily available to consumers in the US. Because dietary supplements are not intended to treat, diagnose, cure, or alleviate the effects of diseases, they do not undergo the drug approval process with the US Food and Drug Administration (FDA).^11^ For dietary supplements, the FDA’s Center for Food Safety and Applied Nutrition is responsible for the agency's oversight of these products, and premarket approval is not required for many ingredients that have historically been in the marketplace. However, new dietary ingredients do require premarket notification with the Center for Food Safety and Applied Nutrition, with supporting information that the ingredient will reasonably be expected to be safe under the conditions of use recommended or suggested in the labeling.

The US National Institutes of Health (NIH), part of the HHS, comprises 27 different institutes and centers that support biomedical research, including research on many aspects of CAM. The National Center for Complementary and Integrative Health, formerly the National Center for Complementary and Alternative Medicine, is the NIH agency with a mission to define, through rigorous scientific investigation, the usefulness and safety of complementary and integrative health interventions and their roles in improving health and health care.^12^ The NIH Office of Dietary Supplements was created to strengthen knowledge and understanding of dietary supplements by evaluating scientific information, stimulating and supporting research, disseminating research results, and educating the public to foster an enhanced quality of life and health for the US population.^13^ Another NIH institute, the National Cancer Institute (NCI), has at least two programs that specifically address traditional medicines. NCI’s Developmental Therapeutics Program has empowered its Natural Products Branch to acquire plants, microbes, and marine organisms through collection contracts encompassing over 25 tropical and subtropical countries worldwide. The NCI Natural Products Repository, which is considered a national resource, includes approximately 80,000 plant samples, 20,000 marine invertebrates and algae, and 16,000 microbes.^14^ The NCI Office of Cancer Complementary and Alternative Medicine is responsible for NCI’s research agenda in CAM as it relates to cancer prevention, diagnosis, treatment, and symptom management.^15^

In the United States, there is currently no standardized, national system for credentialing complementary health practitioners, and the credentials vary significantly from state to state and discipline to discipline.^16^ State and local governments are responsible for deciding what credentials practitioners must have to work in their jurisdiction.

In this context, President Barack Obama and Prime Minister Narendra Modi, in their India-US Joint Statement of January 2015, pledged to encourage dialogue between the United States and India on TM.^17^ In April 2015, the Secretary and Joint Secretary of the Ministry of AYUSH, along with other associates, visited the HHS and NIH to explore the potential for collaboration. As an outcome of this visit, AYUSH and NCI proposed a follow-up meeting as an opportunity for additional discussions. In September 2015, the Joint Secretary of the Ministry of AYUSH participated in the First US-India Health Dialogue in Washington DC,^18^ where both sides agreed that they would collaborate on various aspects of TM, including regulatory assessment and research capacity building. Specifically, the HHS Office of Global Affairs, NCI, and the Ministry of AYUSH agreed to hold a workshop on TM in early 2016 to create a roadmap for AYUSH collaborations between India and the United States and to contribute to health, wellness, and people-centered health care in both nations.

## FIRST INDIA-US WORKSHOP ON TRADITIONAL MEDICINE

The first India-US Workshop on Traditional Medicine (ayushworkshop.in) was held at the National Agricultural Science Complex, New Delhi, India, on March 3 and 4, 2016. The workshop’s inaugural session started with a welcome address by Joint Secretary Jitendra Sharma (Ministry of AYUSH) and was followed by keynote addresses by Ambassador Jimmy Kolker (Assistant Secretary for Global Affairs, HHS), H.K. Pande (Special Secretary, Ministry of Environment, Forest and Climate Change, Government of India), and Ajit Mohan Sharan (Secretary, AYUSH); a special address was provided by US Ambassador to India, Richard Verma, and an address was presented by Shripad Y. Naik (Honorable Minister of State for AYUSH, Government of India). The speakers noted that the goal of this workshop was to discuss the importance of applying rigorous scientific methodologies to the study of traditional Indian medical systems, using evidence derived from such studies to inform both traditional medical practices, appropriately integrating evidence-based traditional practices with modern (Western) medical practices, and making use of the particular strengths that India and the United States can bring to this endeavor.

The workshop was attended by Indian and US policymakers, scientists, academicians, and medical practitioners from various disciplines, including but not limited to natural product chemistry, pharmacology, biochemistry, cancer biology, immunology, cancer prevention, cancer control and population sciences, medical oncology, radiation oncology, integrative oncology, yoga, Ayurveda, Unani, and homeopathy.

The program included specific themes divided into four sessions: (1) Traditional Medicine in the National Cancer Institute, (2) Traditional Medicine in India (introduced by the Ministry of AYUSH), (3) Strength of AYUSH and Promising AYUSH Interventions for Cancer and Other Areas (presented by AYUSH scientists), and (4) Research Presentations on Alternate Systems and Cancer (presented by US scientists). The second day of the workshop was devoted to group discussions on (1) Natural Products Research, (2) AYUSH Systems of Medicine in Cancer: What Holds Promise, and (3) Generating Evidence Toward Market Access for AYUSH Products and Practice.

The remainder of this article summarizes the workshop proceedings; recommendations from the first India-US Workshop on Traditional Medicine, including areas that deserve additional development in cancer research and management; and possible research areas for Indo-US collaboration. The article also highlights developments in the bilateral collaboration since the workshop.

## TM IN THE NCI

**Fig 1:**
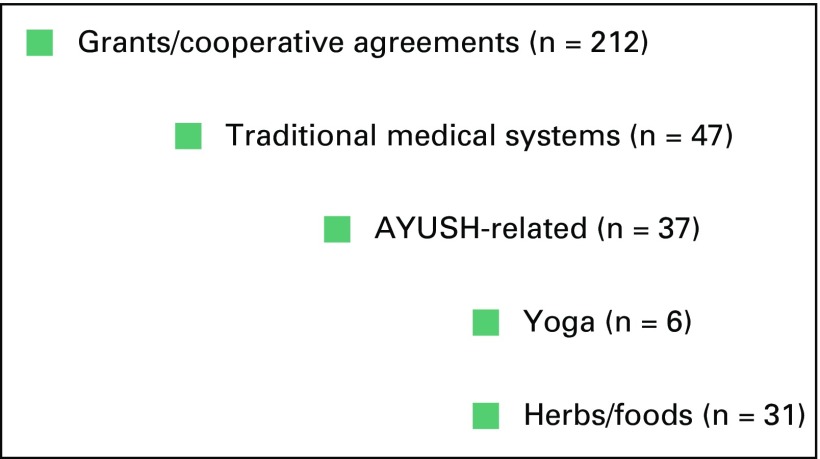
National Cancer Institute’s complementary and alternative medicine research portfolio for fiscal year 2014. AYUSH, Ayurveda, Yoga and Naturopathy, Unani, Siddha, and Homoeopathy.

The presenters in this session were Jeffrey D. White, MD (Director, Office of Complementary and Alternative Medicine, Division of Cancer Treatment and Diagnosis, NCI), Barry R. O’Keefe, PhD (Chief, Natural Products Branch, Division of Cancer Treatment and Diagnosis, NCI), and Ikhlas Khan, PhD (Director, National Center for Natural Products Research, University of Mississippi). White presented an overview of CAM use and research in the United States, with an emphasis on cancer. In fiscal year 2014 (October 1, 2013 to September 30, 2014), NCI supported 212 grants for which some component of the research was relevant to CAM (Fig 1). More than one sixth of these grants involved either yoga or an herb or food associated with Indian culture and TM (Table 1). A grant to Yale University for research on an herbal mixture derived from a traditional Chinese medicine formula, PHY-906, was used as an example of NCI support for investigation of a multiherb product similar to those used in Ayurveda, Siddha, and Unani. PHY-906 is a four-herb formula that has been shown to enhance the therapeutic indices of various classes of anticancer agents in preclinical studies and is now being used in a randomized phase II clinical trial with irinotecan in patients with advanced colorectal cancer.^19^ O’Keefe described the work of NCI’s Natural Products Branch and Molecular Targets Laboratory. The NCI Natural Products Repository is one of the largest and most diverse collections of natural products, housing more than 230,000 extracts. The growth inhibitory effects of these extracts and their fractions are tested in the NCI-60 Human Tumor Cell Line Screen, as well as against discrete cancer-related molecular targets. O’Keefe described how the active components in extracts are isolated, identified, and assessed for their mechanism of action. He discussed the use of analytical techniques, such as liquid chromatography mass spectroscopy–mass spectroscopy to help upgrade the Ayurveda, Siddha, and Unani pharmacopeia and bring it on a par with the US Pharmacopeia.

**Table 1:**
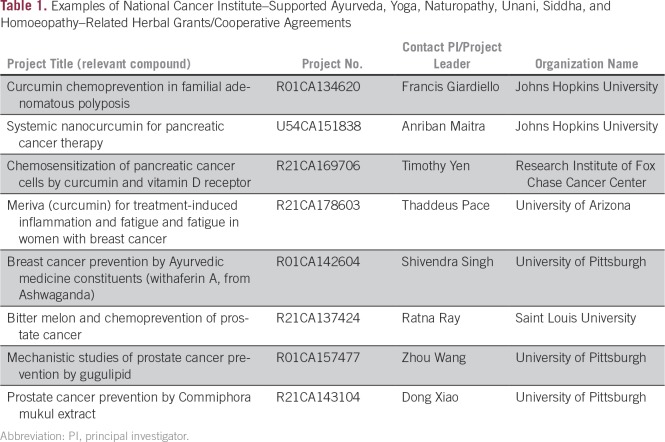
Examples of National Cancer Institute–Supported Ayurveda, Yoga, Naturopathy, Unani, Siddha, and Homoeopathy–Related Herbal Grants/Cooperative Agreements

 Khan explained the US government’s approach to regulating botanical products as either dietary supplements or drugs. Important issues in the regulatory assessments of these products are safety, quality, and efficacy. Several aspects of product quality were examined, including standardization, selection of marker compounds, adulteration, and misidentification. Finally, Khan introduced the Indo-US Center for Research in Indian Systems of Medicine at the University of Mississippi, which has as its mission the facilitation of scientific validation and dissemination of information on Indian systems of medicine through collaborative research.

## TM IN INDIA: MINISTRY OF AYUSH

On behalf of the Ministry of AYUSH, Sharma presented a brief overview of AYUSH and AYUSH research programs, including current collaborations with US institutions. Presentations of promising AYUSH interventions for treating cancer included a collaborative study with the Tata Memorial Centre-Advanced Centre for Treatment Research and Education in Cancer, Mumbai, which has identified nine medicinal plants with potential cytotoxic or cytostatic effects against different types of cancers, including cancers of the oral cavity, lung, ovary, colon, and prostate. In addition, a former patient with cancer shared his experience with treatment through the use of meditation, yoga, and Ayurvedic medicines, along with a vegetarian diet and simple living.

## STRENGTH OF AYUSH AND PROMISING AYUSH INTERVENTIONS FOR CANCER AND OTHER AREAS (PRESENTATIONS BY AYUSH SCIENTISTS)

The main thrust of this session was that AYUSH interventions/therapies have the potential to be used for regression of the disease process in cancer, managing its symptoms and complications, reducing the adverse effects from chemotherapeutic drugs, improving quality of life, increasing the survival period, and preventing recurrences and complications. 

Chitra Mandal, PhD (Indian Institute of Chemical Biology, Kolkata), discussed a new molecule, CM-5, a carbazole alkaloid derived from the plant Murraya koenigii (Rutaceae), commonly known as curry leaf or kari patta, which has shown tumoricidal activity and also increased the efficacy of known cancer drugs in an animal model of various types of cancers at doses that did not cause serious systemic toxicity. CM-5 has also been shown to exhibit antiproliferative activity against several pancreatic cancer cells through apoptosis.^20^ Similarly, a presentation by Subhash Padhye, PhD (Interdisciplinary Science and Technology Research Academy, Pune), demonstrated the potential anticancer therapeutic effects of a Unani intervention, black cumin seeds (Nigella sativa). The major active component of black cumin is thymoquinone, which demonstrates cytotoxic activity against pancreatic cancer cell lines by targeting the prolactin receptor.

The utility of yoga in improving the quality of life in patients with cancer was highlighted through two presentations. R. Nagarathna, MBBS, MD, Medical Director, S-VYASA Deemed University, Bengaluru, and Raghavendra Rao, PhD, Senior Scientist and Head, CAM Program, Health Care Global Enterprises, gave a presentation on the integration of Ayurveda and yoga with conventional Western medicine to relieve the symptoms of cancer, notably, nausea, vomiting, fatigability, alopecia, diarrhea, insomnia, pain, and the adverse effects of chemotherapy, radiation, and surgery. Ramesh Bijlani, MBBS, MD, Emeritus Professor of Physiology, All India Institute of Medical Sciences, highlighted the use of yoga as a potential cancer prevention strategy. G. Geetha Krishnan, BAMS, MD (Ayurveda), Senior Consultant & Head of the Department of Integrative Medicine, Medanta Hospital, discussed his work on the use of yoga and Ayurvedic therapies, such as Panchakarma, to prevent adverse effects of radiotherapy and chemotherapy, including nausea, vomiting, and insomnia.

A pilot study in which metal-based formulations from Ayurveda were used to treat acute promyelocytic leukemia was presented by Vaidya Balendu Prakash of the Vaidya Chandra Prakash Cancer Research Foundation in Dehradun. The study, which was conducted in collaboration with the Central Council for Research in Ayurvedic Sciences, the Ministry of AYUSH, and the All India Institute of Medical Sciences, provided some evidence for consistent and sustained antileukemic effects.

A preclinical study of the anticancer effect of a homeopathic preparation of Thuja occidentalis was presented by Gaurisankar Sa, PhD, Division of Molecular Medicine, Bose Institute, Kolkata. Results from this study suggested that homeopathic medicines prepared from Thuja sulfur and Calcarea carbonica in dynamic or potentized doses may be able to induce apoptosis in cancer cells through a p53-dependent pathway in various cell lines studied. Jayesh V. Sanghvi, MD (Homeopathy), Director, Nature Clinic Super Specialty Center in Chennai, proposed homeopathy as a single therapy or as an add-on therapy for relieving pain and other symptoms and adverse effects of radiotherapy, chemotherapy, and surgery.

 N. Deepa, M.Pharm, PhD, Vice Principal, College of Pharmacy, Chennai, discussed Siddha interventions derived from aqueous extracts of two plants that showed anticancer properties in preclinical studies. These extracts were shown to be cytotoxic to a cancer cell line, apparently through DNA damaging effects. 

Arvind Kulkarni, MD, Director, Radiation Oncology Department, Lady Ratan Tata Medical Center, spoke about integrative oncology and highlighted the functioning of the Ayurveda Centre in the Integrated Cancer Hospital, which has been established in Pune and declared a Centre of Excellence by Ministry of AYUSH. The Centre has done work in evaluating the efficacy of combinations of Ayurvedic drugs in alleviating drug toxicity and improving the quality of life in patients with cancer treated with chemotherapy.^21^

## RESEARCH PRESENTATIONS ON ALTERNATE SYSTEMS OF MEDICINE AND CANCER (PRESENTATIONS BY US SCIENTISTS)

This session provided examples of relevant US research projects and programs. Nagi Kumar, PhD, RD, Director, Cancer Chemoprevention, Moffitt Cancer Center, presented on chemoprevention and treatment of cancers with botanicals. She provided a detailed description of the process of drug development with reference to botanicals and discussed current challenges for targeted therapies in cancer, as well as the advantage of a multitargeted approach through botanicals. Limitations of the present biomarker-based approaches were also described, along with ways to overcome these limitations to facilitate botanical drug development.

Hasan Mukhtar, PhD, Helfaer Professor of Cancer Research, Director and Vice Chair for Research, Department of Dermatology, Wisconsin Medical Sciences Center, presented on natural products for cancer chemoprevention on the basis of lessons learned from animal experiments. Studies exploring potentially effective approaches for cancer control in humans were discussed. Encouraging findings from these prevention studies indicate the need for additional investigations of these and other natural products.

Guido Pauli, Pharm D, PhD, Professor and University Scholar at the University of Illinois at Chicago College of Pharmacy, presented on advancing the holistic approach to traditional plant-based medicine. He elaborated on the need to study natural products both qualitatively and quantitatively, and presented approaches for a more holistic analysis that captures the complex constitution of natural health products. Although marker compounds for natural products are used widely and globally, they should be thoroughly investigated and defined with caution. Validation methodology for chemotaxonomic and bioactive markers as well as potential leads for active pharmaceutical ingredients should be congruent.

## GROUP DISCUSSION

Day 2 of the workshop began with discussions to identify research areas for Indo-US collaboration and to summarize the meeting outcomes. The workshop attendees were divided into three discussion groups: (1) Natural Products Research; (2) AYUSH Systems of Medicine in Cancer: What Holds Promise; and (3) Generating Evidence Toward Market Access for AYUSH Products and Practices.

### Natural Products Research (Group 1)

The natural products research group discussed research methods, training needs, prerequisites for material transfer agreements with US NCI and Indian perspectives, intellectual property rights, and National Biodiversity Authority perspectives on benefit sharing and commercialization.

Several recommendations emerged from these discussions:

- The need to add capacity for conducting research in natural products, including both single herbs and mixtures. Suggestions included building a central repository for cell lines, extracts, and fractions, as well as developing infrastructure for translation of validated research into clinical practice, including incubation of start-up companies.
- The need for harmonization of pharmacopeias from India and Western nations, including the United States. Important for such harmonization is the creation of a centralized laboratory for analytical methodology devoted to standardized quality control for Ayurvedic preparations. Such a facility would lead to the development of standard operating procedures (SOPs) for herbal and herbomineral preparations.
- The proposed enhancement of quality control and standardization in Ayurvedic preparations would better ensure the reproducibility of results among disparate laboratories to increase transferability between institutes and countries. To support such reproducibility studies, the group recommended that the Ministry of AYUSH encourage multicenter clinical studies on interventions for the most prevalent cancers in India and the United States. These studies should use a standard format for data collection, analyses, and management. Open dissemination of the results of such clinical trials and evaluation of their power and reproducibility are critical.
- Additional recommendations included focusing on the strengths of traditional medical systems such as rasayanas (and similar medicinal preparations) and the development of specialized expertise in intellectual property rights.

### AYUSH Systems of Medicine in Cancer: What Holds Promise? (Group 2)

The overall goal of this group was to identify research gaps and ultimately develop steps for generating evidence and moving research to practice. The group discussed research methods, training needs, and next steps. The focus was on epidemiology studies, palliative care, and support studies. The discussion led to the following recommendations:

- Evidence to translate research to practice should be obtained in a culture of collaboration, ensuring communication and cooperation among all stakeholders, including AYUSH, extramural scientists, clinicians, and pharmaceutical/biotechnology companies. AYUSH activities should be well integrated, involving both basic scientists and clinical teams.
- Basic research is needed to generate robust supporting data for claims regarding the integrity, safety, and efficacy of botanicals that are well founded (eg, effects on immunity), to inform the development and implementation of clinical trials.
- There is a critical need to develop guidelines and standard operating procedures to ensure product integrity (standardization, stability), including a clear understanding of biology, molecular targets, safety, and indications for use of AYUSH products.
- Clinical trials should be performed with scientific rigor and include safety and clinical efficacy studies. Focus areas should include adjuvant therapy and remission therapy by AYUSH modalities, benefits from such approaches in the form of personalized medicine, and improvement in quality of life.
- Mechanisms should be established that encourage the articulation of results of TM observations in scientific meetings and their publication in peer-reviewed journals.
- With regard to the training and education of the next generation of TM practitioners, the group felt that joint Indo-US training programs would be mutually beneficial. One particular recommendation was the joint development of protocols for clinical and basic research. Such protocols should consider quantitative clinical parameters.
- Finally, the formation of US-India activity groups was recommended to continue discussions of relevant topics.

### Generating Evidence Toward Market Access for AYUSH Products and Practices (Group 3)

The overall goals of this group were to identify gaps, define the key role of research, discuss approaches to implementing validation studies, consider the role of rigorous clinical trials, and identify areas for collaboration that would ultimately lead to generating sufficient evidence to move research results into clinical practice. Recommendations from this group included the following:

- Recognize the need to advance basic sciences in AYUSH by establishing collaborations among basic, clinical, and population science research teams as well as the private sector and industry.
- Establish guidelines and standards for product integrity in terms of biology, targets, and rigor/reproducibility, including developing SOPs for product development, validation, batch-to-batch consistency, preclinical models, and clinical trials.
- Build centralized Good Manufacturing Practice laboratories, using established manufacturing practices for product development and create a hub for making this information, including cost of production of products, available to all users.
- Establish a regulatory structure to address consistency in standards of research conduct and compliance.
- Articulate and publish findings of studies conducted using rigorous scientific methodology.
- Create cross-disciplinary exchange and scholarships, including a platform for joint India-US conferences and/or symposia, providing training opportunities across and within disciplines.

## RECENT DEVELOPMENTS

After the workshop, the participating agencies agreed to adopt a multidisciplinary approach for moving ahead in the intersection of Indian TM and cancer research. Several meetings have been held with representatives of NCI, HHS, National Institute of Cancer Prevention and Research (NICPR)/Indian Council of Medical Research, the All India Institute of Ayurveda and the Ministry of AYUSH. All India Institute of Ayurveda has been declared the nodal center to coordinate AYUSH cancer research in India. Under the aegis of an MoU signed with NICPR–Indian Council of Medical Research, a Centre for Integrative Oncology has been set up in Noida at NICPR where officers from each AYUSH system are stationed to collate all information related to cancer research done in AYUSH systems. This center will also coordinate international activities, including with the NCI and HHS. The Ministry of AYUSH has also signed memoranda of understanding (MoUs) with the United States Pharmacopeia Convention and Pharmacopeia Commission of Indian Medicine and Homoeopathy; Homoeopathic Pharmacopeia Convention of United States and Central Council for Research in Homeopathy and Pharmacopeia Commission of Indian Medicine and Homoeopathy. This will help in the development and harmonization of pharmacopeias for the traditional systems of medicine. The Ministry of AYUSH is also deputing officers to the National Centre for Natural Products Research, University of Mississippi, for postdoctoral training. A draft MoU between HHS and the Ministry of AYUSH is in the process of finalization, with the aim of facilitating capacity building and joint academic and research collaborations. These progressive developments will help take forward the shared commitments of the two countries in the field of TM.

In conclusion, the workshop served as an important initial engagement for the Ministry of AYUSH, HHS–Office of Global Affairs, and NCI to discuss cooperative activities in areas including research training and research programs. These discussions highlighted the opportunities to develop rigorous scientific portfolios and collaboration, while integrating existing knowledge, clinical best practices, and considering regulatory issues that govern the process of bringing scientific results to the benefit of public health.

The workshop provided a comprehensive understanding of existing gaps, such as inclusion of rational and scientific approaches for drug development; policies and procedures that can help develop milestones/expected outcomes; promotion of collaborative research; implementation of regulatory structures to ensure product reproducibility; and development of procedures and principles for product validation. The workshop also provided the opportunity for NCI and AYUSH researchers to begin engagement that continues as agreements and priorities are formalized.

Several important recommendations emerged from the workshop, such as the need for basic sciences in deepening the understanding of TM; creation of cross discipline exchange/scholarships; development of centers of excellence; centralized laboratories; a platform for joint India-U.S. conferences and symposia to share and acquire knowledge; a hub of standardized information; regulatory structure and compliance; and development of Good Manufacturing Practice facilities. It would seem highly beneficial to link together some of the well-established centers in India for basic, translational, and clinical research with joint projects. Such projects should emphasize product supply and encourage industries to participate in product development. Development of plant biotechnology was another area of interest. Implementing data and procedure optimization via development of SOPs and validation procedures was highly recommended. Development of training programs for TM scientists and medical practitioners, as well as crosstraining opportunities across all relevant disciplines, was also prominent among the recommendations.

The first bilateral workshop provided a strong foundation for AYUSH, NCI, and their partners to work together on implementing the recommendations for better development and promotion of the growth of traditional Indian medicine research, advancing its evidence-based practice, and defining its role in the care of patients with cancer in an integrated fashion with conventional biomedicine. A Centre for Integrative Oncology is in place to coordinate interactions on cancer-related research at the national and international levels. One of the premium institutes of Ayurveda has been designated as a nodal institute for cancer research. Efforts are under way to formalize the collaboration between HHS and AYUSH to advance the research and regulatory capacity of TM.
